# Cross-sectional adherence with the multi-target stool DNA test for colorectal cancer screening in a large, nationally insured cohort

**DOI:** 10.1007/s00384-021-03956-0

**Published:** 2021-05-21

**Authors:** Lesley-Ann Miller-Wilson, Lila J Finney Rutten, Jack Van Thomme, A Burak Ozbay, Paul J Limburg

**Affiliations:** 1 Exact Sciences Corporation, Madison, WI USA; 2 Mayo Clinic, 200 First Street SW, Rochester, MN USA

**Keywords:** Colorectal cancer screening/prevention, Colorectal neoplasms, Early detection of cancer, Mt-sDNA, Insurance

## Abstract

**Purpose:**

Colorectal cancer (CRC) is the second most deadly cancer in the USA. Early detection can improve CRC outcomes, but recent national screening rates (62%) remain below the 80% goal set by the National Colorectal Cancer Roundtable. Multiple options are endorsed for average-risk CRC screening, including the multi-target stool DNA (mt-sDNA) test. We evaluated cross-sectional mt-sDNA test completion in a population of commercially and Medicare-insured patients.

**Methods:**

Participants included individuals ages 50 years and older with commercial insurance or Medicare, with a valid mt-sDNA test shipped by Exact Sciences Laboratories LLC between January 1, 2018, and December 31, 2018 (*n* = 1,420,460). In 2020, we analyzed cross-sectional adherence, as the percent of successfully completed tests within 365 days of shipment date.

**Results:**

Overall cross-sectional adherence was 66.8%. Adherence was 72.1% in participants with Traditional Medicare, 69.1% in participants with Medicare Advantage, and 61.9% in participants with commercial insurance. Adherence increased with age: 60.8% for ages 50–64, 71.3% for ages 65–75, and 74.7% for ages 76 + years. Participants with mt-sDNA tests ordered by gastroenterologists had a higher adherence rate (78.3%) than those with orders by primary care clinicians (67.2%). Geographically, adherence rates were highest among highly rural patients (70.8%) and ordering providers in the Pacific region (71.4%).

**Conclusions:**

Data from this large, national sample of insured patients demonstrate high cross-sectional adherence with the mt-sDNA test, supporting its role as an accepted, noninvasive option for average-risk CRC screening. Attributes of mt-sDNA screening, including home-based convenience and accompanying navigation support, likely contributed to high completion rates.

## Introduction

Colorectal cancer (CRC) is the second leading cause of cancer deaths and fourth most diagnosed cancer in the USA [1], representing an ongoing public health concern, with estimates of 149,500 incident and 52,980 fatal cases in 2021 [2]. Average-risk CRC screening can favorably impact the CRC public health burden by identifying patients with premalignant or localized malignant neoplasia for earlier, more effective intervention. National organizations such as the United States Preventive Services Task Force (USPSTF) in their 2021 Final Recommendation Statement and the American Cancer Society (ACS) recommend lowering the age for average-risk CRC screening (beginning at the age of 45 years) using one of several equally endorsed test options, including the multi-target stool DNA assay [3–5] (mt-sDNA; marketed as Cologuard®; Exact Sciences, Madison, WI). Since receiving approval from the US Food and Drug Administration in August 2014, the mt-sDNA assay has been prescribed by over 200,000 providers and completed by more than 4 million patients nationwide.

Between 2015 and 2018, estimated CRC screening rates increased overall by 4.2%, from 61.7 to 65.9% according to National Health Interview Survey data [6, 7]. The same National Health Interview Survey (NHIS) data showed that for those who completed screening, 4.1% used mt-sDNA, and use was consistent across demographic subgroups such as sex, age, and race/ethnicity, with no apparent disparities. The number of people screened with the mt-sDNA test further increased in 2019, with 1.7 million patients successfully screened using mt-sDNA testing [8]. Despite the COVID-19 disruption, the at-home mt-sDNA test with its built-in navigation component for patients and providers and door-to-door shipping is uniquely positioned to sustain colorectal cancer screening efforts, potentially tempering the backlog of CRC screening delays and corresponding late-stage disease.

One critical component of effective CRC screening programs is patient adherence with completing the selected test option. The practical effectiveness of available screening strategies may be reduced by suboptimal adherence to screening recommendations [9]. Discouragingly, prior research has demonstrated relatively low completion rates for other stool-based CRC screening tests [9–14], and several recently conducted trials have revealed differences in CRC screening completion rates by test modality [9–11, 14]. These differences in completion rates may be exacerbated by multiple factors, such as socioeconomic status [15, 16] and race [17, 18]. Given the differences between endoscopic, radiologic, and stool-based screening strategies, accurate understanding of test-specific patient adherence is critical for population-, provider-, and payor-level discussions.

To date, analyses of mt-sDNA adherence have demonstrated high cross-sectional adherence rates (71%) but have been limited to Medicare beneficiaries to minimize the influence of insurance variability on test completion rates [19]. Given the growing adoption of mt-sDNA screening by commercial insurance plans, nearly all insured (> 94%) average-risk patients now have access to mt-sDNA screening with no out-of-pocket costs [20]. Thus, additional real-world assessment of mt-sDNA test adherence in non-Medicare patients is both feasible and timely. Here, we evaluated cross-sectional mt-sDNA test completion in a fully insured population and identified associated factors, to better inform shared decision-making, quality monitoring, and comparative effectiveness studies related to average-risk CRC screening.

## Methods

Aggregate laboratory data from Exact Sciences Laboratories LLC (ESL; Madison, WI), the sole-source national laboratory for mt-sDNA testing, were retrospectively reviewed as part of ongoing laboratory quality management processes and in compliance with the Health Insurance Portability and Accountability Act. Per Mayo Clinic Institutional Review Board (IRB) criteria, this study was deemed as exempt from review.

### Study population, design, and data acquisition

Eligible study participants included individuals who met the following criteria: ages 50 years and older, covered by commercial insurance or Medicare, with a valid mt-sDNA test shipped to the order-specified address from ESL between January 1, 2018 and December 31, 2018. At the time of the study, the USPSTF recommendation was to start screening at 50 years old. Valid mt-sDNA test shipments were defined as having all information required for ESL to analyze the sample and report a positive or negative test result. Deidentified data referent to available patient (sex, age), provider (specialty, practice location), and test order features (testing status, time to completion) were sourced from the ESL internal data systems. Of note, patient race/ethnicity is listed as an optional field on the mt-sDNA order form and was additionally collected when available.

**Fig. 1:**
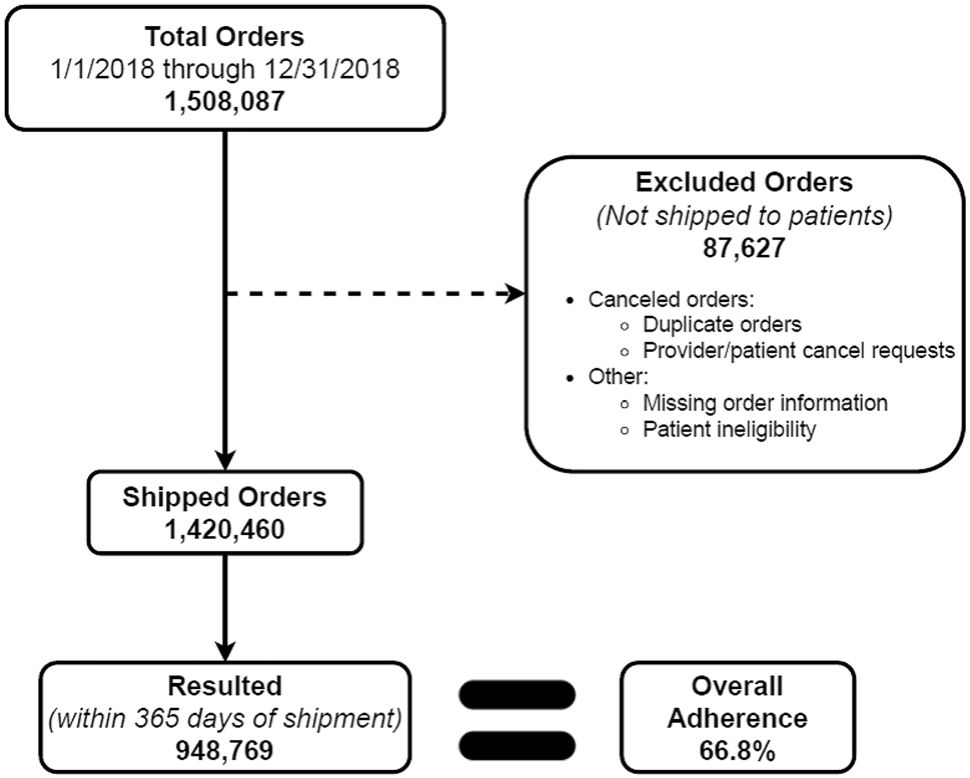
Study cohort—attrition flowchart

Information from ESL internal data systems regarding order characteristics was initially collected for 1,508,087 patients. Inclusion and exclusion criteria (including provider/patient cancellations, duplicate orders, missing information, and reasons for ineligibility) were utilized to comprise the final analysis cohort consisting of 1,420,460 patients. The cohort attrition flow chart outlines that cross-sectional adherence was defined as the percentage of eligible participants who successfully completed the test within 365 days of the shipment date (Fig. 1).

Sociodemographic characteristics including income and education are not tracked in the ESL test order data system. Therefore, these data were ascertained at the residential zip code level from public data sources, including the United States Census and American Community Surveys database [21–23].

### Statistical analysis

Descriptive statistics were used to describe the baseline characteristics of the study population overall and by insurance type. Counts and percentages were used to describe population-level statistics while distribution statistics (mean and median) were provided, where appropriate, to further describe the study populations’ time to adherence, overall adherence, and age. Descriptive statistics were principally stratified by patient insurance type. Distribution statistics relating to skewness and other appropriate tests (e.g., chi-square test) were used as needed to describe the study population.

Given that the study population comprises the entirety of mt-sDNA patients during this time (*N* = 1,420,460), point estimates and *p*-value testing do not provide necessary context or support better comparison. In essence, very small differences, even if significant, are likely to arise due to the population size though do not reflect meaningful differences among populations. In contrast to significance tests, effect size is independent of sample size, while statistical significance can be dependent upon both sample size and effect size [24, 25]. To avoid confounding the meaning of significance due to such a large sample size, we decided not to report two-sided 95% confidence intervals for point estimates or two-sided *p*-values for subgroup comparisons.

## Results

Of 1,420,460 patients who met the study criteria (Fig. 1), 61.2% were female with a mean age of 64.9 years. Age distributions were 50–64 years (47.3%), 65–75 years (40.0%), and 76 + years (12.7%), with the majority of the cohort residing in urban areas (70.4%). Of those who specified a race/ethnic category, the majority of participants were white, although most participants (85.6%) did not provide this optional information (Table 1). Within the study population, 46.1% had commercial insurance coverage, 33.5% had Traditional Medicare coverage, and 20.4% were covered by Medicare Advantage (Table 2). Patients residing in zip codes with above average Bachelor’s degree attainment rates (35.0% of all adults above 25 in the USA had a Bachelor’s degree as of 2018) [22] accounted for 31.2% of all shipments. Patients in zip codes with a median income greater than the national median ($33,706 per the American Community Survey conducted in 2018) [23] accounted for 43.3% of mt-sDNA test orders. Geographically, the highest adherence in the study population was observed for those dwelling in highly rural locations: urban (66.0%), rural (68.5%), and highly rural (70.8%) (Table 3). Most mt-sDNA test orders were placed by primary care clinicians (72.2%), followed by nurse practitioners and physician assistants (19.3%), gastroenterologists (3.2%), and obstetrics and gynecologists (1.8%), with the remaining mt-sDNA test orders placed by other specialties (3.5%) (Table 4).

**Table 1:** Study population demographics by insurance coverage type

Category (% of cohort)	Insurance type			
	Commercial	Traditional Medicare	Medicare advantage	Total
Age				
50–64, *n* (%)	573,126 (87.5)	56,953 (12.0)	41,501 (14.3)	671,580 (47.3)
65–75, *n* (%)	71,187 (10.9)	307,007 (64.5)	190,617 (65.8)	568,811 (40.0)
76^+^, *n* (%)	10,546 (1.6)	112,111 (23.5)	57,412 (19.8)	180,069 (12.7)
Totals	654,859	476,071	289,530	1,420,460
Sex				
Male, *n* (%)	260,436 (39.8)	178,860 (37.6)	110,708 (38.2)	550,004 (38.7)
Female, *n* (%)	394,174 (60.2)	296,962 (62.4)	178,732 (61.7)	869,868 (61.2)
Unspecified, *n* (%)	249 (0)	249 (0)	90 (0)	588 (0)
Race				
White (%)	87,625 (13.4)	60,967 (12.8)	35,393 (12.2)	183,985 (13.0)
Black or African American (%)	6999 (1.1)	4304 (0.9)	4298 (1.5)	15,601 (1.1)
American Indian/Alaska Native (%)	244 (0)	176 (0)	103 (0)	523 (0)
Asian (%)	2844 (0.4)	1034 (0.2)	820 (0.3)	4698 (0.3)
Native Hawaiian or other Pacific Islander (%)	190 (0)	119 (0)	79 (0)	388 (0)
Not specified (%)	556,957 (85.0)	409,471 (86.0)	248,837 (86.0)	1,215,265 (85.6)
Geography				
Urban (%)	471,962 (72.1)	317,426 (66.7)	210,140 (72.6)	999,528 (70.4)
Rural (%)	165,099 (25.2)	141,133 (29.6)	72,247 (25.0)	378,479 (26.7)
Highly rural (%)	17,798 (2.7)	17,512 (3.7)	7143 (2.5)	42,453 (3.0)

**Table 2:** Cross-sectional adherence by patient factors

Patient-level factors	Cross-sectional adherence											
	Total			Commercial (46.1%)			Traditional Medicare (33.5%)			Medicare advantage (20.4%)		
	Completed/shipped	%	TTA	Completed/shipped	%	TTA	Completed/shipped	%	TTA	Completed/shipped	%	TTA
All eligible study patients	948,769/1,420,460	66.8	22	405,652/654,859	61.9	24	343,135/476,071	72.1	21	199,982/289,530	69.1	21
Age, years												
50–64	408,516/671,580	60.8	24	351,850/573,126	61.4	24	32,106/56,953	56.4	24	24,560/41,501	59.2	24
65–75	405,662/568,811	71.3	21	46,642/71,187	65.5	22	225,931/307,007	73.6	21	133,089/190,617	69.8	21
76^+^	134,591/180,069	74.7	20	7160/10,546	67.9	20	85,098/112,111	75.9	20	42,333/57,412	73.7	20
Sex												
Male	369,655/550,004	67.2	21	162,896/260,436	62.5	23	129,790/178,860	72.6	20	76,969/110,708	69.5	20
Female	578,748/869,868	66.5	22	242,611/394,174	61.5	25	213,183/296,962	71.8	21	122,954/178,732	68.8	22
Not specified	366/588	62.2	26	145/249	58.2	27	162/249	65.1	27	59/90	65.6	21
Race												
White	118,016/183,985	64.1	23	52,668/87,625	60.1	25	42,056/60,967	69.0	22	23,292/35,393	65.8	22
Black or African American	7,972/15,601	51.1	24	3373/6999	48.2	26	2228/4304	51.8	23	2371/4298	55.2	23
American Indian/Alaska Native	290/523	55.4	24	145/244	59.4	24	88/176	50.0	23	57/103	55.3	23
Asian	2593/4698	55.2	23	1451/2844	51.0	24	667/1034	64.5	23	475/820	57.9	23
Native Hawaiian or other Pacific Islander	233/388	60.1	23	101/190	53.2	23	83/119	69.7	25	49/79	62.0	21
Not specified	819,665/1,215,265	67.4	22	347,914/556,957	62.5	24	298,013/409,471	72.8	21	173,738/248,837	69.8	21

*TTA* time to adherence (median days)

**Table 3:** Cross-sectional adherence by zip code population factors

Zip code population statistics	Cross-sectional adherence											
	Total			Commercial			Traditional Medicare			Medicare advantage		
	Completed/ordered	%	TTA	Completed/ordered	%	TTA	Completed/ordered	%	TTA	Completed/ordered	%	TTA
Employment status—unemployment rate												
Above national rate (70.9%)	667,427/1,006,915	66.3	21	276,955/451,104	61.4	22	241,674/337,704	71.6	21	148,798/218,107	68.2	21
Below national rate (29.1%)	281,342/413,545	68.0	22	128,697/203,755	63.2	23	101,461/138,367	73.3	21	51,184/71,423	71.7	22
Education level—bachelor’s degree rate												
Above national rate (31.2%)	292,479/443,321	66.0	23	119,511/195,280	61.2	24	103,612/146,796	70.6	22	69,356/101,245	68.5	22
Below national rate (68.8%)	656,290/977,139	67.2	21	286,141/459,579	62.3	22	239,523/329,275	72.7	21	130,626/188,285	69.4	21
Median income												
Above national rate (43.3%)	419,161/614,364	68.2	22	192,521/303,687	63.4	24	147,879/199,270	74.2	22	78,761/111,407	70.7	22
Below national rate (56.7%)	529,608/806,096	65.7	22	213,131/351,172	60.7	22	195,256/276,801	70.5	21	121,221/178,123	68.1	21
Geography												
Urban (70.4%)	659,291/999,528	66.0	23	289,431/471,962	61.3	24	226,679/317,426	71.4	22	143,181/210,140	68.1	22
Rural (26.7%)	259,403/378,479	68.5	22	104,651/165,099	63.4	23	103,155/141,133	73.1	22	51,597/72,247	71.4	21
Highly rural (3.0%)	30,075/42,453	70.8	22	11,570/17,798	65.0	23	13,301/17,512	76.0	21	5204/7143	72.9	22

*TTA* time to adherence (median days)

**Table 4:** Cross-sectional adherence by provider-level factors

Provider-level factors	Cross-sectional adherence											
	Total			Commercial			Traditional Medicare			Medicare advantage		
	Completed/ordered	%	TTA	Completed/ordered	%	TTA	Completed/ordered	%	TTA	Completed/ordered	%	TTA
Specialty												
GI (3.2%)	35,381/45,204	78.3	20	8,830/12,106	72.9	22	19,296/23,920	80.7	20	7255/9178	79	20
OB/GYN (1.8%)	16,149/25,597	63.1	24	10,817/17,220	62.8	26	3660/4855	75.4	22	1672/3522	47.5	23
Primary care (72.2%)	689,393/1,025,331	67.2	22	288,894/462,109	62.5	24	249,634/345,462	72.3	21	150,865/217,760	69.3	21
NP/PA (19.3%)	174,441/274,694	63.5	22	83,070/141,027	58.9	24	57,167/83,264	68.7	21	34,204/50,403	67.9	21
Other (3.5%)	33,405/49,634	67.3	22	14,041/22,397	62.7	24	13,378/18,570	72	21	5986/8667	69.1	21
Practice location (region)												
East North Central	200,228/301,581	66.4	21	93,746/151,818	61.7	23	63,003/89,215	70.6	20	43,479/60,548	71.8	20
East South Central	67,801/101,257	67	20	25,800/41,222	62.6	22	24,040/33,879	71	19	17,961/26,156	68.7	20
Mid-Atlantic	112,383/171,083	65.7	23	49,104/79,746	61.6	25	38,759/55,519	69.8	21	24,520/35,818	68.5	22
Mountain	63,508/95,063	66.8	23	23,060/36,321	63.5	26	23,203/30,882	75.1	22	17,245/27,860	61.9	23
New England	44,068/67,562	65.2	24	21,710/35,799	60.6	27	14,606/20,885	69.9	22	7752/10,878	71.3	23
Pacific	56,805/79,522	71.4	25	23,117/34/519	67	27	26,858/35,750	75.1	23	6830/9253	73.8	24
South Atlantic	229,657/342,664	67	21	93,501/152,993	61.1	23	89,721/123,081	72.9	20	46,435/66,590	69.7	21
West North Central	67,400/96,140	70.1	22	30,671/46,937	65.3	23	25,984/34,687	74.9	20	10,745/14,516	74	21
West South Central	106,707/165,239	64.6	22	44,860/75,362	59.5	24	36,878/52,044	70.9	21	24,969/37,833	66	22
Puerto Rico and US Territories	212/349	60.7	24	83/142	58.5	25	83/129	64.3	24	46/78	59	23

*GI* gastroenterology, *GYN* gynecology, *NP* nurse practitioner, *OB* obstetrics, *PA* physician assistant, *TTA* time to adherence (median days)

The overall cross-sectional adherence rate for the entire study population was 66.8% (948,769/1,420,460 patients received and completed the mt-sDNA test and had a valid result) with a median time-to-adherence (TTA) of 22 days. The difference in adherence between males and females was small (67.2 vs. 66.5%, respectively). Cross-sectional adherence was highest in patients with Traditional Medicare coverage (72.1%) and lowest in patients with commercial insurance coverage (61.9%). Adherence increased with age: 50–64 years (60.8%), 65–75 years (71.3%), and 76 + years (74.7%) overall, and within each of the insurance types (Table 2). Relative to those with commercial insurance, increases in adherence by age were more pronounced among those with Traditional Medicare or Medicare Advantage plans, who demonstrated lower rates of adherence among those 50–64 compared to those with commercial insurance (Table 2). Moreover, adherence was 73.6% for Traditional Medicare patients ages 65–75.

Statistically, cross-sectional adherence with the mt-sDNA test was not shown to be associated with level of education or median income of the zip code wherein patients resided. Adherence was 66.0% in zip codes with an above average Bachelor’s degree attainment rate compared with 67.2% in zip codes with a below average Bachelor’s degree attainment rate. Similarly, adherence was 68.2% compared to 65.7% in zip codes with median incomes above and below the national median, respectively (Table 3).

Adherence also varied among ordering provider specialty and practice location. Patients with mt-sDNA tests ordered by gastroenterologists exhibited a higher adherence rate (78.3%) than those with orders placed by primary care clinicians (67.2%), nurse practitioners and physician assistants (63.5%), obstetricians and gynecologists (63.1%), and other specialties (67.3%). Adherence rates were highest among patients with ordering providers in the Pacific region (71.4%) and West North Central region (70.1%). Contrastingly, the lowest adherence was observed in the Mid-Atlantic region (65.7%), New England (65.2%), West South-Central region (64.6%), and Puerto Rico/US Territories (60.7%) (Table 4).

## Discussion

Results from this retrospective study of a large, insured population demonstrate relatively high cross-sectional adherence with the mt-sDNA test for CRC screening. Adherence rates were similar between males and females and increased with age. Of note, area-level education and median income of patients’ residential zip codes were not significantly associated with differences in adherence rates. Consistent with previous observations in Medicare patients [19], adherence rates were also higher for orders placed by gastroenterologists compared to other provider specialties. Geographically, the greatest adherence was observed among those living in highly rural areas; however, that population was only 3% of the overall cohort. Although GIs ordered the fewest mt-sDNA tests, the rate of adherence to recommendations by disease specialists may relate in part to more detailed discussion during encounters regarding the importance of completing CRC screening; albeit, this speculative interpretation requires further evaluation.

With nearly 1.5 million participants included in the study population, our data contributes to the emerging literature regarding mt-sDNA test adherence in the clinical setting, although in a younger and larger cohort than seen previously [19]. Overall adherence approached 67%, with meaningful differences observed between age groups. The highest rate was seen in the 76 + years subgroup having Traditional Medicare coverage. Adherence also increased with age, from 60.8% in those 50–64 to 74.7% in patients ≥ 76 + years, while Traditional Medicare patients ages 65–75 exhibited 73.6% adherence.

The mt-sDNA test is offered in conjunction with patient and provider navigation support (available 24 h per day, 365 days per year, with translation services in over 240 languages). Previous studies have shown that patient navigation has a beneficial effect on patient health behavior, demonstrating increased CRC screening rates [19, 26, 27]. The amalgamation of test and navigation services was shown to be successful in a separate study of Medicare beneficiaries where individuals who were previously non-adherent with CRC screening were offered the mt-sDNA test, resulting in 88% completion and 96% diagnostic colonoscopy exam follow-up for those with a positive mt-sDNA result [28]. This intricate feature adds inherent value to the mt-sDNA test at no additional cost to the payor, health system, patient, or provider, while also improving the probability of test completion compared with no intervention [19–29]. This information may be helpful to consider as a guiding input variation for CRC modeling studies to investigate comparative effectiveness between screening modalities.

There is general agreement concerning the effectiveness of colorectal cancer screening; however, in contrast to other organ sites, major guideline groups have endorsed more than one test option for average-risk CRC screening in order to maximize engagement. Informed health care provider discussions and screening decisions are better guided by implementation considerations that can help patients select the testing option they are most likely to complete, and this factor is essential for reducing the number of CRC-related deaths in the USA. Prior research has demonstrated low completion rates for other CRC screening tests [9, 10], and existing literature on repeat screening with other non-mt-sDNA stool-based screening tests suggests a need for improvement [9–11, 30]. A more sensitive test, such as mt-sDNA, is less susceptible to drops in adherences rates. Mt-sDNA has a higher single application sensitivity for all stages of CRC, which distinguishes it from single marker fecal occult blood tests (FIT/gFOBT) [31]. The 10,000-person mt-sDNA pivotal study by Imperiale et al. [31] reported 92% sensitivity for CRC (vs 74% for FIT). This performance difference held true in receiver operating characteristic (ROC) analysis, regardless of the cutoff chosen for FIT. Higher one-time test sensitivity is relevant given that FIT adherence to recommended screening frequency can drop off substantially after the first cycle of annual testing [32].

As an integral part of the screening process, the follow-up colonoscopy to a positive non-colonoscopy CRC screening exam is becoming increasingly appreciated in the literature. Follow-up colonoscopy after a positive first-line test is necessary for complete and effective CRC screening [33, 34]. Failure to, and delay of follow-up after a positive stool-based test has been associated with increased risk of later stage CRC and CRC mortality [33, 35–37]. Doubeni et al. found that patients failing to follow-up on abnormal FIT results face a sevenfold higher risk of dying of CRC than those who complete the follow-up process [37].

Differences have also been revealed in CRC screening completion rates by modality [13, 14]. In a study by Rutten et al. [14] among residents of Olmsted County, MN, eligible and due for CRC screening, it was revealed that CRC screening incidence rates remained stable from 2016 to 2018, while test-specific rates for mt-sDNA significantly increased. Moreover, the study revealed that adherence with follow-up colonoscopy within 6 months after a positive stool-based test was significantly higher among patients who underwent mt-sDNA screening versus FIT/FOBT (84.9% vs 42.6%, respectively). At real-world (imperfect) adherence rates of 40% for annual FIT and 70% for triennial mt-sDNA derived from a critical assessment of meta-analyses and retrospective cross-sectional data [19, 38] (in systems using FIT without a navigation program) [39], modeling illustrates that the number of LYG and reductions in CRC incidence and mortality were higher for triennial mt-sDNA [40].

Several geographic variations in adherence aligned with the currently reported US percentage of adults being up-to-date with CRC screening tests by state [41], with the highest rates seen in the Pacific and West North Central regions in our study, although adherence was lower in the New England and Middle Atlantic regions where up-to-date screening hovers around ≥ 70% and ≥ 65%, respectively.

Additionally, practice specialty was associated with higher cross-sectional adherence. Although gastroenterologists ordered fewer tests, adherence was lower among primary care providers. The observed difference in adherence by specialty could be related to various provider factors (strength of screening recommendation, patient perception of specialty-related knowledge), patient factors (attitude concerning referral/follow-up completion), or a combination. It is also likely that patients who present to a gastroenterologist bring with them specific concerns which may serve as motivation to screen. Although test adherence rates differed by practice, recent literature has shown widespread awareness of the 2018 ACS CRC guidelines among primary care providers [42]. A 2020 survey study by the Montana Cancer Control Program revealed that fecal DNA testing has become much more popular since 2016, with more than a third (35.2%) of primary care providers reporting discussing it with patients in 2020 compared to only 4% in 2016 [43]. The same report from the Montana Department of Public Health and Human Services highlighted that in 2020 the fecal DNA test was the 3rd highest ranked CRC screening test prescribed with 86% of ordering providers considering the test effective. Future CRC screening studies can be tailored to garner information on provider ordering characteristics to better inform screening strategy, assess prescription differences, and develop provider education initiatives.

We would like to acknowledge several limitations of our study. First, there was limited reporting of ethnicity in this study population, with less than 15% of patients specifying a racial category. The unavailability of such data prohibited us from examining an important demographic aspect associated with screening adherence; thus, the differences observed among race/ethnicity were not overly considered due to a large percentage of participants not providing the optional information. Patient and provider factors analyzed in our study were limited to those characteristics that could be captured from the existing laboratory database. A more detailed assessment of factors suspected to influence mt-sDNA screening adherence, such as socioeconomic status along with a closer look at social determinants of health, will benefit future investigations. Secondly, we relied on determination of the mt-sDNA ordering clinician to define CRC average-risk status. Although indicated for mt-sDNA, we assumed that each provider appropriately followed test eligibility indications before prescribing, and we did not have access to sufficient data to confirm risk status for all participants in this real-world study. Thus, a percentage of off-label use can be logically assumed. Lastly, given the relative recency of mt-sDNA availability and adoption in clinical practice, we did not assess longitudinal adherence for the current study. Mt-sDNA test adherence was only evaluated on a cross-sectional basis with a review of associated patient and provider factors over a 1-year study period. Further evaluation of longitudinal adherence with triennial mt-sDNA screening is anticipated in future studies.

## Conclusions

Novel data from this large retrospective cohort of both Medicare and commercially insured patients revealed favorable adherence to CRC screening with the mt-sDNA assay test among eligible patients who met the study criteria, having a valid mt-sDNA test shipped to their order-specified address from ESL between January 1, 2018, and December 31, 2018. Innovative features of the mt-sDNA test such as the accompanying patient navigation system, together with its noninvasive approach and feasibility, likely impacted test completion rates. The highest adherence rates were observed among those covered by Medicare and for tests ordered by gastroenterologists. Our findings contribute valuable real-world data to the existing evidence base and provide clinical decision-makers with supportive information to recommend a guideline-endorsed CRC screening strategy, with the goal of increasing adherence through test completion. Future investigations examining longitudinal completion rates in accordance with national guidelines, as well as more detailed analyses of associations with socioeconomic, race/ethnicity, and other demographic factors on mt-sDNA test completion rates, are anticipated to complement this study.

## Data Availability

Study data are available upon reasonable request.
